# Limited-stage small cell lung cancer: Outcomes associated with prophylactic cranial irradiation over a 20-year period at the Princess Margaret Cancer Centre

**DOI:** 10.1016/j.ctro.2021.06.009

**Published:** 2021-07-08

**Authors:** Michael Yan, Tzen S. Toh, Patricia E. Lindsay, Jessica Weiss, Katrina Hueniken, Christy Yeung, Vijithan Sugumar, Dixon Pinto, Tony Tadic, Alexander Sun, Andrea Bezjak, John Cho, Srinivas Raman, Meredith Giuliani, Fabio Ynoe Moraes, Geoffrey Liu, Andrew J. Hope, Benjamin H. Lok

**Affiliations:** a Department of Oncology, Cancer Centre of Southeastern Ontario, Queen’s University, Kingston, ON, Canada; b The Medical School, University of Sheffield, Sheffield, United Kingdom; c Division of Medical Oncology and Hematology, Princess Margaret Cancer Centre, Toronto, ON, Canada; d Radiation Medicine Program, Princess Margaret Cancer Centre, Toronto, ON, Canada; e Department of Radiation Oncology, University of Toronto, Toronto, ON, Canada; f Department of Biostatistics, Princess Margaret Cancer Centre, Toronto, ON, Canada; g Department of Laboratory Medicine and Pathology, University of Toronto, Toronto, ON, Canada; h Department of Physiology and Pharmacology, Western University, London, ON, Canada; i Faculty of Health Sciences, McMaster University, Hamilton, ON, Canada; j Department of Medical Biophysics, University of Toronto, Toronto, ON, Canada; k Institute of Medical Science, University of Toronto, Toronto, ON, Canada

**Keywords:** Small-cell lung cancer, Prophylactic cranial irradiation, Limited-stage

## Abstract

**Background & purpose:**

Prophylactic cranial irradiation (PCI) is recommended for limited-stage small-cell lung cancer (LS-SCLC) patients with good response to concurrent chemoradiation. We report our institution’s 20-year experience with this patient population and associated clinical outcomes.

**Materials & methods:**

A retrospective cohort of consecutive LS-SCLC patients treated with curative intent chemoradiation at our institution (1997–2018) was reviewed. Overall survival (OS) was calculated using the Kaplan-Meier method, and significant covariates determined by the Cox proportional hazards model. Covariates predictive of PCI were determined using Fisher's exact test and the Mann-Whitney test. Brain failure risk (BFR) was calculated using the cumulative incidence method treating death as a competing event. Treatment cohorts (historic vs. contemporary) were stratified by the median year of diagnosis (2005).

**Results:**

A total of 369 patients with LS-SCLC were identified, of which 278 patients were notionally PCI eligible. PCI was given to 196 patients (71%). Younger age was associated with PCI utilization (p < 0.001). PCI utilization rates did not change between the historic and contemporary treatment era (p = 0.11), whereas magnetic resonance imaging (MRI) use at baseline and follow-up became more prevalent in the contemporary era (p = <0.001). On multivariable analysis, PCI utilization was associated with improved OS (HR 1.88, 95% CI 1.32–2.69) and decreased BFR (HR 4.66, 95% CI 2.58–8.40). Patients who had MRI follow-up had a higher incidence of BFR (HR 0.35, 95% CI 0.18–0.66) in multivariable analyses.

**Conclusions:**

For LS-SCLC patients at our institution, PCI is more frequently utilized in younger patients, and the utilization rate did not change significantly over the past 20 years. PCI was independently associated with improved OS and lower BFR. Omission of PCI in LS-SCLC patients should not be routinely practiced in the absence of further prospective data.

## Highlights

• Prophylactic cranial irradiation (PCI) was more commonly used in younger patients.
• PCI utilization rates did not change throughout our 20-year institutional experience.
• PCI was associated with improved OS and lower brain metastasis risk, independent of MRI follow-up or era of treatment.
• For LS-SCLC patients with good thoracic response, PCI remains the standard-of-care.

## Introduction

Small-cell lung cancer (SCLC) has a high propensity for brain metastases, with 10% of patients having intracranial disease at presentation. As such, prophylactic cranial irradiation (PCI) is recommended for limited-stage small-cell lung cancer (LS-SCLC) patients with good thoracic response to initial treatment. PCI has been shown to improve absolute brain metastasis control by 25.3% and overall survival by as much as 5.4% at 3 years [1]. However, PCI utilization rates vary, with rates as low as 43% and 55% in various single institutional series attributable to various patient and physician concerns regarding lack of perceived benefit, patient fitness, or neurocognitive toxicities [2], [3].

Recently, a phase III randomized trial demonstrated a lack of OS benefit with PCI for patients with extensive-stage small-cell lung cancer (ES-SCLC) who underwent regular magnetic resonance imaging (MRI) surveillance [4]. Whether this finding could be extrapolated to the LS-SCLC population is unknown and is a point of contention as to whether PCI remains beneficial in the MRI era [5], [6].

In the absence of randomized evidence, we aimed to report the outcomes of PCI in our large institutional cohort of LS-SCLC patients over the past two decades.

## Materials & methods

### Clinical characteristics

We retrospectively reviewed all LS-SCLC patients treated with radiotherapy (RT) at the Princess Margaret Cancer Centre from 1997 to 2018. Electronic medical records (EMR) were queried to extract details of baseline characteristics, systemic therapy, RT, surveillance, and clinical outcomes. All patients had pathological confirmation of their disease diagnosis. To determine temporal trends, patients were stratified into two cohorts based on the median date of diagnosis: (A) historic cohort (before 17th November 2005) and (B) contemporary cohort (on or after 17th November 2005). The median date of diagnosis was chosen in order to achieve similar sample sizes in each arm to allow for comparative power.

Clinical stage was determined using the 8th edition of the American Joint Committee on Cancer (AJCC) staging system [7]. Staging investigations were performed with computed tomography (CT), bone scan, and/or positron emission tomography (PET); brain imaging consisted of CT or MRI. Comorbidities were calculated using a modified Charlson Comorbidity Index (mCCI) score: congestive heart failure (CHF) − 2, chronic obstructive pulmonary disease (COPD) − 1, liver disease − 2, hemiplegia − 2, dementia − 2, renal disease − 1, diabetes − 1, and HIV/AIDS – 4 [8]. This study was conducted with approval from the institutional research ethics board.

### Treatment characteristics

Patients were CT-simulated using a helical planning scan or a 4D planning scan. Thoracic RT was delivered by intensity-modulated radiotherapy (IMRT), volumetric modulated arc therapy (VMAT), 3D conformal, or opposed pair techniques. Thoracic RT was given as 40 Gy in 15 daily fractions (equivalent dose in 2 Gy fractions with an α/β ratio of 10 [EQD2_10_] = 42 Gy), 45 Gy given in 30 fractions twice daily (EQD2_10_ = 43 Gy), 60 Gy in 30 daily fractions, or 66 Gy in 33 daily fractions. The use of 4D-CT and image guided radiotherapy (IGRT) varied over time. The institutional policy was for radiotherapy to be given concurrently with platinum-based chemotherapy and etoposide, usually with the first or second cycle. Otherwise, sequential chemotherapy followed by radiation was given.

PCI was offered to all patients deemed to have a good thoracic response (complete or partial response) to chemoradiation. A shared decision-making approach was employed for patients who had baseline cognitive comorbidities or decreased performance status. The typical PCI dose was 25 Gy in 10 daily fractions (EQD2_10_ = 26 Gy) and was delivered using lateral parallel opposed fields with multileaf collimator (MLC) shielding of the lens and oral cavity. Patients who had stable or progressive disease after chemotherapy and radiation, completed less than 4 cycles of chemotherapy, received less than 40 Gy of thoracic RT, or only received one treatment modality were excluded from the current analysis.

Brain follow-up practices varied, from no imaging, to interval CT or MRI scans. There was no universal institutional policy implemented over the 20-year study period; follow-up schedules were at the discretion of the treating physician. Patients were defined to have had brain imaging follow-up if at least one cranial scan was performed post treatment completion, measured from the end of thoracic chemoradiotherapy. Brain relapses were typically treated with whole brain radiotherapy (WBRT) or, occasionally, stereotactic radiosurgery (SRS).

### Endpoints and statistical analysis

Baseline characteristics were summarized using descriptive statistics. Predictors of PCI utilization were summarized using the Fisher exact test for the categorical variables and the Mann-Whitney for the continuous variables. The Mann-Whitney test was used in our analyses given that age may not be normally distributed, and that SCLC patients tend to be older, hence skewing the distribution of the data.

Overall survival (OS) was calculated from the date of pathological diagnosis to the date of death from any cause or censored at the last clinical visit. OS curves were estimated using the Kaplan-Meier method, with significant covariates determined utilizing the Cox proportional hazards regression model. Brain failure risk (BFR) was calculated from the date of pathological diagnosis and modelled using the Fine and Gray subhazard distribution method; deaths from any cause were treated as a competing event. The significance level was set to 0.05, with all tests being two-sided. Statistical analyses were carried out using R (version 4.0.2) [9].

## Results

### Patient demographics and treatment characteristics

**Fig. 1:**
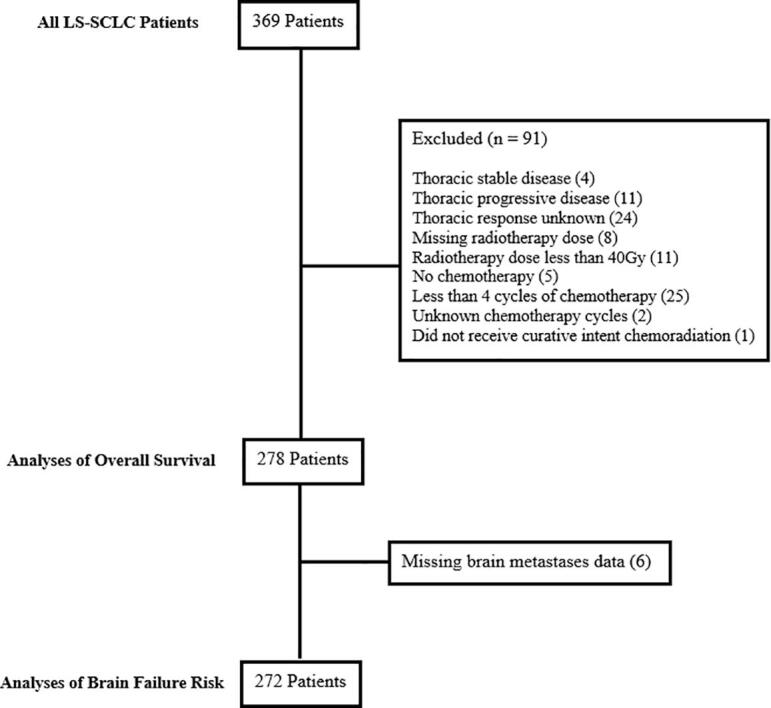
Consort diagram depicting the final analysis cohort of eligible LS-SCLC patients.

A total of 369 LS-SCLC patients treated with radiation from 1997 to 2018 were identified. After exclusions, 278 patients were eligible for inclusion in our analysis (Fig. 1). The median follow-up was 22.3 months (interquartile range [IQR]: 13.8–49.9). Table 1 describes the baseline cohort characteristics stratified by PCI utilization. The median patient age was 66 years (range 39–91), and 160 patients (58%) were male. Most patients were Eastern Cooperative Oncology Group (ECOG) performance status 0–1 at the time of diagnosis (86%) and received concurrent chemoradiotherapy (83%) and were able to complete at least 4 cycles of chemotherapy (87%). Younger age (<66 years) was significantly associated with PCI utilization (p < 0.001).

**Table 1:** Baseline patient and treatment characteristics.

Characteristic	Total Cohort (n = 278)	PCI Administered (n = 196)*	PCI Not Administered (n = 81)*	p-value
Median age (years, range)	65.6 (38.7–90.6)	64.2 (38.7–85.0)	69.7 (44.9–90.6)	<0.001
Stage (n, %)				0.58
I	27 (11)	21 (12)	5 (7)	
II	28 (11)	20 (11)	8 (11)	
III	192 (78)	135 (77)	57 (81)	
Unknown	31	20	11	
ECOG status (n, %)				1.00
0/1	235 (86)	165 (86)	69 (86)	
2/3	38 (14)	27 (14)	11 (14)	
Unknown	5	4	1	
mCCI Score (n, %)				0.40
0	176 (65)	128 (67)	47 (61)	
1+	93 (35)	63 (33)	30 (39)	
Unknown	9	5	4	
Paraneoplastic Syndrome (n, %)				0.20
No	253 (93)	181 (94)	71 (90)	
Yes	19 (7)	11 (6)	8 (10)	
Unknown	6	4	2	
Pre treatment brain imaging (n, %)				0.12
CT	75 (28)	52 (28)	22 (27)	
MRI	174 (65)	124 (67)	50 (62)	
None	17 (6)	8 (4)	9 (11)	
Unknown	12	12	0	
Radiotherapy Dose				0.43
40 Gy/15	183 (66)	123 (63)	59 (73)	
45 Gy/30 BID	82 (29)	64 (33)	18 (22)	
60 Gy/30	3 (1)	2 (1)	1 (1)	
66 Gy/33	3 (1)	2 (1)	1 (1)	
Other	7 (3)	5 (3)	2 (2)	
Chemotherapy (n, %)				0.29
Concurrent	230 (83)	158 (81)	71 (88)	
Sequential	46 (17)	36 (19)	10 (12)	
Unknown	2	2	0	
Brain Surveillance (n, %)				0.18
MRI	155 (56)	115 (59)	40 (49)	
No-MRI	123 (44)	81 (41)	41 (51)	
Brain Relapse Salvage Therapy (n, %)**				1.00
WBRT	58 (88)	29 (88)	29 (88)	
SRS	8 (12)	4 (12)	4 (12)	

Abbreviations: ECOG, Eastern Cooperative Oncology Group; PCI, prophylactic cranial irradiation; mCCI, modified Charlson Comorbidity Index; WBRT, whole brain radiotherapy; SRS, stereotactic radiosurgery

* PCI status unknown for one patient

** Only patient with brain relapse and received salvage therapy were included

The historic cohort consisted of 139 patients and the contemporary cohort had 139 patients. Among the historical cohort, 45% of patients were staged pretreatment with an MRI brain, 66% of patients received PCI, and 40% received MRI brain follow-up. In contrast, 84% of patients within the contemporary cohort received MRI brain staging, 75% of patients received PCI, and 71% received MR brain follow-up. The distribution of PCI utilization did not change significantly (p = 0.11) over the two eras. However, both MRI staging and MRI follow-up were more common in the contemporary era (p < 0.001). Additionally, more patients were treated with 45 Gy/30 BID in the contemporary era, and generally, were of poorer ECOG status (ECOG 0–1 in 79% vs. 93% p < 0.001) (Table S1).

### Outcomes

#### The effect of treatment era on outcomes

When adjusting for the period of treatment, we found that the use of PCI was associated with improved OS. In the historic cohort, the 5-year OS was 32% (95% CI: 24–44) for those who received PCI and 19% (95% CI: 10–35) for those who did not receive PCI. In the contemporary cohort, these respective survivals were 32% (95% CI: 23–45) and 28% (95% CI: 15–52; Fig. 2**A**).

**Fig. 2:**
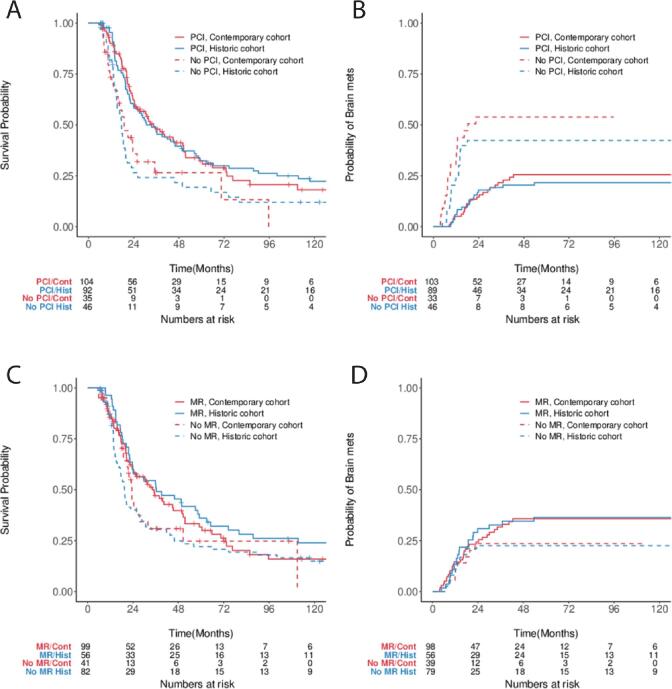
OS and BFR Stratified by Treatment Era. Kaplan-Meier and cumulative incidence function estimates stratified by treatment era and PCI for (A) OS and (B) BFR; and by MR follow-up utilization for (C) OS and (D) BFR.

Similarly, we found that PCI improved BFR risk independent of the treatment era. In the historic cohort, the 5-year BFR rate for patients who received PCI was 22% (95% CI: 13–31) compared to 41% (95% CI: 26–56) for those who did not receive PCI. In the contemporary cohort, the BFR rates were 26% (95% CI: 17–35) and 56% (95% CI: 36–72) respectively (Fig. 2**B**).

There was no difference in OS associated with the use of MRI follow-up, regardless of diagnosis date (Fig. 2**C**). There was a higher observed BFR for patients who received post-treatment brain MRI imaging, in both the contemporary and historic cohorts, with a 5-year BFR rate of 36% for those who received MRI follow-up, and 22 and 24% in the historical and contemporary cohorts respectively, for those who did undergo not MRI follow-up (Fig. 2**D**).

#### PCI utilization and MRI Follow-up

Overall, 56% of patients received at least one MR brain post-treatment. The median MRI follow-up for the entire cohort was 14.6 months (IQR: 6.2–31.5). Of the patients who received MRI follow-up, the majority (52%) received 3 or more scans.

**Fig. 3:**
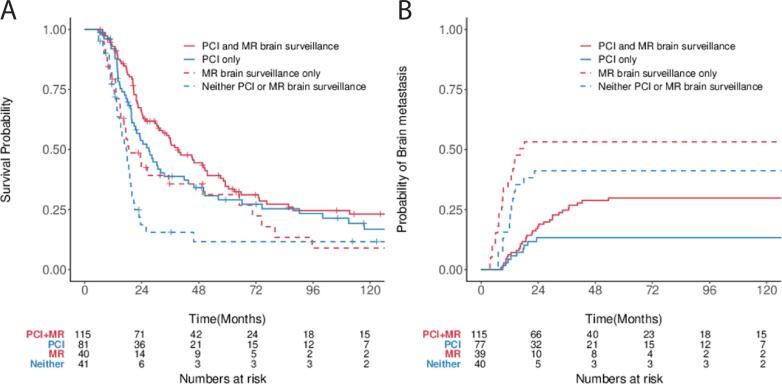
OS and BFR Stratified by PCI and MR Follow-up. Kaplan-Meier and cumulative incidence function estimates stratified by PCI and MR follow-up utilization for (A) OS and (B) BFR.

The 5-year OS for patients who received PCI and MRI brain follow-up, PCI alone, MRI follow-up alone, and neither were 35% (95% CI: 26–45), 29% (95% CI: 20–42), 31% (95% CI: 19–53), and 12% (95% CI: 4–31) respectively (Fig. 3**A**). The 5-year BFR for patients for these same cohorts was 30% (95% CI: 21–39), 13% (95% CI: 6–23), 53% (95% CI: 36–68), and 41% (95% CI: 25–57; Fig. 3**B**).

On univariable analysis, PCI use (HR 1.77: 95% CI, 1.31–2.40), younger age (HR 1.03, 95% CI: 1.01–1.04), and MRI follow-up (HR 1.32, 95% CI, 1.00–1.75) were significantly associated with improved OS. In regard to BFR, PCI use (HR 2.93, 95% CI, 1.85–4.63) was associated with significantly lower BFR. Multivariable regression was performed for both endpoints adjusting for age, stage, mCCI score, cohort era, and ECOG status. PCI use (HR 1.88, 95% CI: 1.32–2.69) and younger age (HR 1.03, 95% CI: 1.01–1.05) remained significantly associated with OS, while PCI use (HR 4.66, 95% CI: 2.58–8.40) and no MRI follow-up (HR 0.35, 95%CI: 0.18–0.66) were associated with lower BFR (Table 2).

**Table 2:** Overall survival and brain relapse-free survival.

	Overall Survival				Brain Failure Risk			
	Univariate Analysis		Multivariate Analysis		Univariate Analysis		Multivariate Analysis	
Covariates	HR (95% CI)	p-value	HR (95% CI)	p-value	HR (95% CI)	p-value	HR (95% CI)	p-value
PCI (no vs. yes [ref])	**1.77 (1.31**–**2.40)**	<0.001*	**1.88 (1.32**–**2.69)**	<0.001*	**2.93 (1.85**–**4.63)**	<0.001*	**4.66 (2.58**–**8.40)**	<0.001*
Age at diagnosis	**1.03 (1.01**–**1.04)**	<0.001*	**1.03 (1.01**–**1.05)**	0.0016*	1.00 (0.97–1.02)	0.90	0.97 (0.95–1.01)	0.11
Stage		0.062		0.095		0.13		0.15
(III vs. I [ref])	1.50 (0.91–2.49)		1.50 (0.89–2.53)		3.08 (1.02–9.27)		3.19 (0.98–10.41)	
(II vs. I [ref]	0.92 (0.47–1.8)		0.95 (0.48–1.88)		3.21 (0.92–11.24)		2.63 (0.67–10.29)	
ECOG (2–3 vs. 0–1 [ref])	1.30 (0.88–1.94)	0.19	1.16 (0.73–1.84)	0.53	1.26 (0.67–2.37)	0.47	1.67 (0.83–3.40)	0.15
CCI Score	0.98 (0.81–1.18)	0.80	0.93 (0.75–1.15)	0.49	1.05 (0.77–1.43)	0.77	1.15 (0.88–1.51)	0.30
Diagnosis Date (historical vs. contemporary [ref])	1.03 (0.78–1.37)	0.83	1.11 (0.79–1.57)	0.53	0.88 (0.56–1.38)	0.58	0.98 (0.56–1.73)	0.95
Brain Imaging Follow Up (No-MRI vs. MRI [ref])	**1.32 (1.00**–**1.75)**	0.049*	1.15 (0.83–1.59)	0.40	0.62 (0.38–1.00)	0.052	**0.35 (0.18**–**0.66)**	0.0013*

Abbreviations: ECOG, Eastern Cooperative Oncology Group; PCI, prophylactic cranial irradiation; CCI, Charlson Comorbidity Index.

* Statistically significant associations.

### Brain metastasis salvage treatment

Information about brain metastasis initial salvage treatment was available for 66 patients; of these 8 patients received SRS and the remaining patients (n = 58) received WBRT as initial salvage therapy. The number of patients that received SRS was higher in the contemporary cohort (n = 7) compared to the historical cohort (n = 1). Of the patients that received WBRT, 32 were in the historic cohort and 26 were in the contemporary cohort. Half of these patients received prior PCI, with an even distribution for those who received SRS and WBRT (Table 1).

**Fig. 4:**
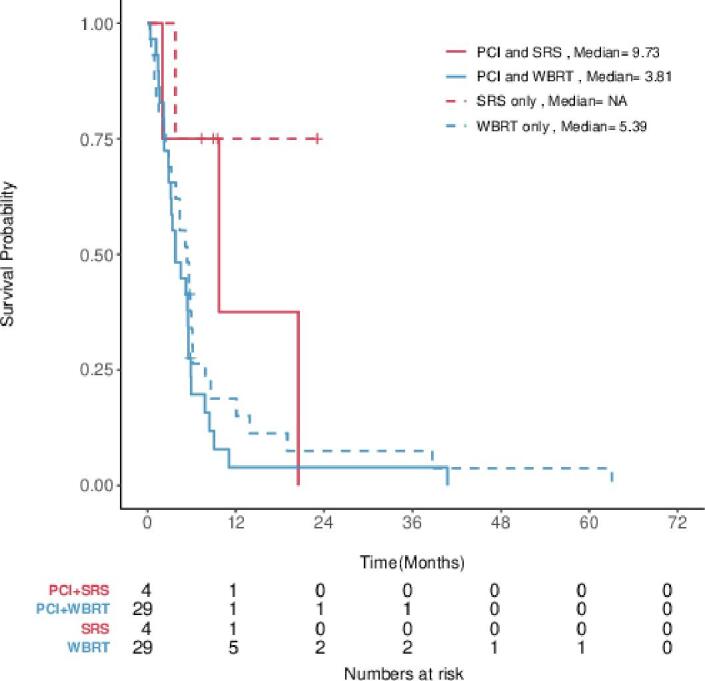
OS stratified by brain salvage technique and PCI. Kaplan-Meier estimates for OS stratified by SRS versus WBRT in patients who did and did not receive PCI.

When stratified by brain salvage technique and PCI, patients that received PCI and SRS had a median OS of 9.73 months (95% CI, 2.04-NA) and those who received PCI and WBRT had a median OS of 3.81 months (95% CI, 3.19–5.56) (Fig. 4). From the date of first detection of brain metastasis, there is a significant difference in OS between the use of SRS and WBRT (HR 3.38, 95% CI, 1.12–9.42). The median OS for those who received SRS and WBRT is 15.2 months (95% CI, 9.7-NA) and 4.9 months (95% CI, 3.4–5.7), respectively.

## Discussion

The benefits of PCI in reducing the incidence of brain metastases in SCLC patients have long been established by several randomized trials from the late 20th century [10], [11], [12], [13]. The survival benefits were later demonstrated in the seminal individual patient *meta*-analysis by Auperin et al, in which an absolute OS benefit of 5.4% at 3 years was observed with PCI [1]. Since then, PCI has been the *de facto* standard of care in this LS-SCLC patient population who have a good thoracic response.

Despite the advantages in intracranial control and survival, there is practice heterogeneity in the recommendation of PCI to all eligible patients. A US national survey of 1231 oncologists in the late 1990s reported that 74% of their respondents would recommend PCI in LS-SCLC patients. Of these respondents, a further 67% of them would only recommend PCI after complete response to initial therapy [14]. It should be noted however, that this survey was carried forth prior to the publication of the Auperin *meta*-analysis [1], yet despite this the majority of oncologists recommended PCI in LS-SCLC with good thoracic response. A contemporary survey was conducted in 2016 with 52 Canadian radiation oncologists; only 52% responded by stating they would offer PCI to LS-SCLC patients if there was any evidence of response to chemoradiation. Various factors were taken into consideration when offering PCI to patients, of which baseline neurocognition, performance status, and tolerance of previous treatment were particularly important [15]. Finally, recent survey responses from members of the International Association for the Study of Lung Cancer in 2018 revealed that 88% and 50% of their 295 respondents, a majority of which were radiation or medical oncologists, would recommend PCI to a 50-year-old patient and a 70-year-old patient, respectively [16].

Unsurprisingly, analyses of modern practice patterns do not reflect the regular utilization of PCI in eligible patients, which is in line with survey observations. A Japanese pattern of care study reported that only 8.6% of their 139 LS-SCLC patients received PCI [17]. Previous large, single-institution studies in the United States and Canada have shown that PCI was delivered in 55–60% of LS-SCLC patients [3], [18]. Our analysis is consistent with these latter studies, in that 71% of notionally eligible patients received PCI.

Common reasons for PCI omission have been previously reported. Patient refusal due to risk of neurotoxicity was the most common reason (38%) given in an institutional analysis from the Memorial Sloan Kettering Cancer Center [3], as well a previous analysis at Princess Margaret Cancer Centre (53%) [18]. The association of PCI utility and neurocognitive decline is well documented in the literature from analyses of prospective randomized trials [19], [20], [21], [22], resulting in complex discussions of risks and benefits with patients to determine the best management course. Physician bias also appears to play a role in PCI decision making; thoracic oncologists who would accept the treatment themselves were most likely to recommend PCI to their patients [16], [23]. In the current study, younger age was the only factor significantly associated with PCI utilization. This is consistent with a previous report, in which patients over the age of 65 were almost 10% less likely to receive PCI [18]. Population level analyses also corroborate decreased utilization of PCI in elderly patients [24].

Perhaps one of the most critical considerations in the contemporary clinical applicability of the Auperin *meta*-analysis is that the study conditions do not reflect modern LS-SCLC patient management; brain MRI was not readily utilized in the included trials. The more recent Japanese randomized trial of PCI in ES-SCLC has sparked controversy in the utility of PCI in the MRI era [4], [5]. In this phase III trial, patients with ES-SCLC were randomized to PCI versus MRI surveillance at 3-month intervals for up to a year then at 6-month intervals until year 2. All trial patients received pre-treatment MR brain staging. Subsequently, the literature has been mixed in regard to the observed OS benefit of PCI in the modern era.

Some retrospective series have not observed a benefit of PCI in subsets of LS-SCLC patients. Early stage and complete thoracic response have been observed to not be associated with survival or brain relapse benefit with the addition of PCI [25], [26], [27]. Two studies looked specifically at patients who had undergone MR brain staging and did not observe a difference in brain metastasis incidence or OS with and without PCI [6], [28].

Conversely, several institutional series support the OS benefit of PCI in the contemporary era for LS-SCLC patients. Patients treated at MD Anderson Cancer Center achieved a significant benefit in OS (HR 0.73, p = 0.001) and brain metastasis risk (HR 0.54, p = 0.002) with the use of PCI. The only subset they reported that did not benefit from PCI, were patients > 70 years old [29]. Similarly, a series from Memorial Sloan Kettering Cancer Center also reported PCI to be significantly associated with improved OS on multivariable analysis (HR 0.67, p = 0.01) [26]. A contemporary Chinese series reported a decrease in brain metastasis (HR 0.24, p < 0.001) incidence as well as mortality (HR 0.60, p < 0.001) with the use of PCI [30]. The results of a recent systematic review and *meta*-analysis of 7 randomized trials showed a significant decrease of brain metastasis incidence (HR 0.45, p < 0.001) as well prolonged OS (HR 0.81, p < 0.001) with the use of PCI. However, the improvement in OS was not evident in the subgroup analysis of patients that received post thoracic chemoradiotherapy CT or MR imaging [31].

Our results support the OS benefits of PCI and are independent of the treatment era. The benefits are substantial, with a nearly 2-fold increase in survival and 4-fold decrease in the development of brain metastases as determined from our multivariate regression models. We did not find an association with OS for patients followed by MRI. The magnitude of effect estimates are consistent with previous series [26], [29], [30]. Interestingly, we note that patients who received MRI based follow-up had a 3-fold higher risk of observed brain failure compared to those who did not; this ostensibly represents the increased sensitivity of MRI in detecting early brain metastases in comparison to other techniques or the ability of patients to tolerate regular follow-up imaging. Nevertheless, this increase in MRI detected brain metastases did not translate into a survival difference after multivariable adjustment likely due to statistical power limitations.

In regard to salvage therapy, we found that there was a significant difference in the OS of patients who received SRS versus WBRT. This corroborates the recent observations of FIRE-SCLC, a multi-institutional cohort study, in which patients treated with SRS were found to have a longer median OS compared to those treated with WBRT (6.5 vs. 5.2 months) that was maintained after propensity score adjustment [32].

Strengths of this study include a large cohort size, granular covariate details, as well as an institutional experience that spans two decades. This allowed for the observation of temporal trends such as the increased use of MR staging and surveillance in the modern era, as well as no significant change in PCI utilization for LS-SCLC patients at our institution. Additionally, our follow-up was robust, with a median of 22 months, and half of patients having a follow-up duration ranging from 14 to 50 months.

Our study is inherently limited by its nature of being a single-institution, retrospective study. The collected data may be prone to selection bias, and a minority of data were incomplete. We also caution the interpretation of the effect of MRI follow-up observed, given that our definition only requires ≥ 1 post treatment MRI scan to have been performed at any time post treatment completion with imaging intervals at the discretion of the clinician. This does not reflect current MRI surveillance practices, in which asymptomatic patients typically receive an MRI every 3 months.

Nevertheless, the current ASTRO practice guidelines recommend PCI for LS-SCLC patients with good response to concurrent chemoradiation who are under 70 and have a good performance status. For patients with poor performance status, older age, or comorbidities, clinical practice guidelines also recommend shared decision-making in regards to the decision to undergo PCI or MRI surveillance while considering patient- and disease-specific characteristics [33]. At our institution, we adhere to these recommendations in that PCI is routinely offered to eligible LS-SCLC patients; although we routinely encourage a shared decision-making approach in which the benefits and risks of MRI brain surveillance and PCI are discussed with the patient.

The results of the current study support the benefits of PCI to improve OS and decrease brain metastasis risk. We eagerly await the results of prospective randomized trials where LS-SCLC patients are eligible to enroll, such as the SWOG S1827 MAVERICK (SWOG S1827) trial comparing PCI to MR surveillance alone (NCT04155034). Additionally, randomized trials of novel brain RT techniques including hippocampal-avoidance PCI are being investigated in the NRG-CC003 trial (NCT02635009) to assess potential improvement in neurocognitive deficits associated with PCI, while maintaining OS and BFR benefit.

PCI was more commonly used in younger patients and utilization did not change through our 20-year institutional experience. PCI was consistently associated with improved OS and lower brain metastasis risk. This was independent of MRI follow-up use or era of treatment. For LS-SCLC patients with good thoracic response, PCI is the standard-of-care and the routine omission of PCI will require forthcoming randomized prospective data.

## Data availability statement

All data and statistical code are available to be shared upon request.

## Funding

None.

## Disclosures

AJH discloses research funding from Elekta. MG discloses research funding from Eli Lilly, and is on the advisory board for AstraZeneca and Bristol Myers Squibb. BHL discloses research funding and honoraria from AstraZeneca, and research funding from Pfizer. No other authors have any pertinent disclosures.

## Declaration of Competing Interest

The authors declare that they have no known competing financial interests or personal relationships that could have appeared to influence the work reported in this paper.

## References

[1] Aupérin A., Arriagada R., Pignon J.-P., Le Péchoux C., Gregor A., Stephens R.J. (1999). Prophylactic cranial irradiation for patients with small-cell lung cancer in complete remission. Prophylactic Cranial Irradiation Overview Collaborative Group. N Engl J Med.

[2] Koh M., Song S.Y., Jo J.H., Park G., Park J.W., Kim S.S. (2019). The value of prophylactic cranial irradiation in limited-stage small cell lung cancer: should it always be recommended?. Radiat Oncol J.

[3] Lok B.H., Ma J., Foster A., Perez C.A., Shi W., Zhang Z. (2017). Factors influencing the utilization of prophylactic cranial irradiation in patients with limited-stage small cell lung cancer. Adv Radiat Oncol.

[4] Takahashi T., Yamanaka T., Seto T., Harada H., Nokihara H., Saka H. (2017). Prophylactic cranial irradiation versus observation in patients with extensive-disease small-cell lung cancer: a multicentre, randomised, open-label, phase 3 trial. Lancet Oncol.

[5] Rusthoven C.G., Kavanagh B.D. (2017). Prophylactic cranial irradiation (PCI) versus active MRI surveillance for small cell lung cancer: the case for equipoise. J Thorac Oncol.

[6] Pezzi T.A., Fang P., Gjyshi O., Feng L., Liu S., Komaki R. (2020). Rates of overall survival and intracranial control in the magnetic resonance imaging era for patients with limited-stage small cell lung cancer with and without prophylactic cranial irradiation. JAMA Netw Open.

[7] Rami-Porta R, Bolejack V, Giroux DJ, Chansky K, Crowley J, Asamura H, et al. The IASLC Lung Cancer Staging Project: The New Database to Inform the Eighth Edition of the TNM Classification of Lung Cancer. Journal of Thoracic Oncology 2014;9:1618–24. https://doi.org/10.1097/jto.0000000000000334. 10.1097/JTO.0000000000000334 25436796

[8] Quan H., Li B., Couris C.M., Fushimi K., Graham P., Hider P. (2011). Updating and validating the Charlson comorbidity index and score for risk adjustment in hospital discharge abstracts using data from 6 countries. Am J Epidemiol.

[9] R Core Team. R: A Language and Environment for Statistical Computing n.d.

[10] Aroney R.S., Aisner J., Wesley M.N., Whitacre M.Y., Van Echo D.A., Slawson R.G. (1983). Value of prophylactic cranial irradiation given at complete remission in small cell lung carcinoma. Cancer Treat Rep.

[11] Ohonoshi T., Ueoka H., Kawahara S., Kiura K., Kamei H., Hiraki Y. (1993). Comparative study of prophylactic cranial irradiation in patients with small cell lung cancer achieving a complete response: a long-term follow-up result. Lung Cancer.

[12] Arriagada R., Le Chevalier T., Borie F., Riviere A., Chomy P., Monnet I. (1995). Prophylactic cranial irradiation for patients with small-cell lung cancer in complete remission. J Natl Cancer Inst.

[13] Gregor A, Cull A, Stephens RJ, Kirkpatrick JA, Yarnold JR, Girling DJ, et al. Prophylactic cranial irradiation is indicated following complete response to induction therapy in small cell lung cancer: results of a multicentre randomised trial. United Kingdom Coordinating Committee for Cancer Research (UKCCCR) and the European Organization for Research and Treatment of Cancer (EORTC). Eur J Cancer 1997;33:1752–8. 10.1016/s0959-8049(97)00135-4 9470828

[14] Cmelak A.J., Choy H., Shyr Y.u., Mohr P., Glantz M.J., Johnson D.H. (1999). National survey on prophylactic cranial irradiation: differences in practice patterns between medical and radiation oncologists. Int J Radiat Oncol Biol Phys.

[15] Shahi J., Wright J.R., Gabos Z., Swaminath A. (2016). Management of small-cell lung cancer with radiotherapy—a pan-Canadian survey of radiation oncologists. Curr Oncol.

[16] Robin T.P., Sannes T.S., Spring Kong F.-M., Mornex F., Hirsch F.R., Rusthoven C.G. (2018). Physician Bias in prophylactic cranial irradiation decision making—an opportunity for a patient decision aid. Clinical Lung Cancer.

[17] Uno T., Sumi M., Ishihara Y., Numasaki H., Mitsumori M., Teshima T. (2008). Changes in patterns of care for limited-stage small-cell lung cancer: results of the 99–01 patterns of care study—a nationwide survey in Japan. Int J Radiat Oncol Biol Phys.

[18] Giuliani M., Sun A., Bezjak A., Ma C., Le L.W., Brade A. (2010). Utilization of prophylactic cranial irradiation in patients with limited stage small cell lung carcinoma. Cancer.

[19] Gondi V., Paulus R., Bruner D.W., Meyers C.A., Gore E.M., Wolfson A. (2013). Decline in tested and self-reported cognitive functioning after prophylactic cranial irradiation for lung cancer: pooled secondary analysis of Radiation Therapy Oncology Group randomized trials 0212 and 0214. Int J Radiat Oncol Biol Phys.

[20] Le Péchoux C., Dunant A., Senan S., Wolfson A., Quoix E., Faivre-Finn C. (2009). Standard-dose versus higher-dose prophylactic cranial irradiation (PCI) in patients with limited-stage small-cell lung cancer in complete remission after chemotherapy and thoracic radiotherapy (PCI 99–01, EORTC 22003–08004, RTOG 0212, and IFCT 99–01): a randomised clinical trial. Lancet Oncol.

[21] Le Péchoux C., Laplanche A., Faivre-Finn C., Ciuleanu T., Wanders R., Lerouge D. (2011). Clinical neurological outcome and quality of life among patients with limited small-cell cancer treated with two different doses of prophylactic cranial irradiation in the intergroup phase III trial (PCI99-01, EORTC 22003–08004, RTOG 0212 and IFCT 99–01). Ann Oncol.

[22] Sun A., Bae K., Gore E.M., Movsas B., Wong S.J., Meyers C.A. (2011). Phase III trial of prophylactic cranial irradiation compared with observation in patients with locally advanced non-small-cell lung cancer: neurocognitive and quality-of-life analysis. J Clin Oncol.

[23] Johnson S.B., Decker R.H. (2018). Prophylactic Cranial Irradiation Versus Surveillance: Physician Bias and Patient-centered Decision-making. Clin Lung Cancer.

[24] Damhuis R.A.M., Senan S., Belderbos J.S. (2018). Usage of prophylactic cranial irradiation in elderly patients with small-cell lung cancer. Clin Lung Cancer.

[25] Farris M.K., Wheless W.H., Hughes R.T., Soike M.H., Masters A.H., Helis C.A. (2019). Limited-stage small cell lung cancer: is prophylactic cranial irradiation necessary?. Pract Radiat Oncol.

[26] Wu A.J., Gillis A., Foster A., Woo K., Zhang Z., Gelblum D.Y. (2017). Patterns of failure in limited-stage small cell lung cancer: Implications of TNM stage for prophylactic cranial irradiation. Radiother Oncol.

[27] Mamesaya N., Wakuda K., Omae K., Miyawaki E., Kotake M., Fujiwara T. (2018). Efficacy of prophylactic cranial irradiation in patients with limited-disease small-cell lung cancer who were confirmed to have no brain metastasis via magnetic resonance imaging after initial chemoradiotherapy. Oncotarget.

[28] Ozawa Y., Omae M., Fujii M., Matsui T., Kato M., Sagisaka S. (2015). Management of brain metastasis with magnetic resonance imaging and stereotactic irradiation attenuated benefits of prophylactic cranial irradiation in patients with limited-stage small cell lung cancer. BMC Cancer.

[29] Farooqi A.S., Holliday E.B., Allen P.K., Wei X., Cox J.D., Komaki R. (2017). Prophylactic cranial irradiation after definitive chemoradiotherapy for limited-stage small cell lung cancer: Do all patients benefit?. Radiother Oncol.

[30] Qiu G., Du X., Zhou X., Bao W., Chen L., Chen J. (2016). Prophylactic cranial irradiation in 399 patients with limited-stage small cell lung cancer. Oncol Lett.

[31] Yin X., Yan D., Qiu M., Huang L., Yan S.-X. (2019). Prophylactic cranial irradiation in small cell lung cancer: a systematic review and meta-analysis. BMC Cancer.

[32] Rusthoven C.G., Yamamoto M., Bernhardt D., Smith D.E., Gao D., Serizawa T. (2020). Evaluation of first-line radiosurgery vs whole-brain radiotherapy for small cell lung cancer brain metastases: the FIRE-SCLC cohort study. JAMA Oncol.

[33] Simone C.B., Bogart J.A., Cabrera A.R., Daly M.E., DeNunzio N.J., Detterbeck F. (2020). Radiation therapy for small cell lung cancer: an ASTRO clinical practice guideline. Pract Radiat Oncol.

