# Supplementary material for: Limited-stage small cell lung cancer: Outcomes associated with prophylactic cranial irradiation over a 20-year period at the Princess Margaret Cancer Centre
Source: Clin Transl Radiat Oncol. 2021 Jul 8;30:43–9. doi: 10.1016/j.ctro.2021.06.009 (PMC8282904; doi:10.1016/j.ctro.2021.06.009)
Supplement: Supplementary data 1 [file mmc1.docx]

**Supplementary Information**

| Characteristic | Total Cohort (n = 278) | Diagnosis before median (n = 139) | Diagnosis after median (n = 139) | p-value |
| --- | --- | --- | --- | --- |
| Median age (years, range) | 65.4 (38.7-90.6) | 64.7 (38.7-88.0) | 66.6 (40.8-90.6) | 0.026 |
| PCI  Yes  No  Unknown | 196 (71)  81 (29)  1 | 92 (66)  47 (34)  0 | 104 (75)  34 (25)  1 | 0.11 |
| Stage (n, %)  I  II  III  Unknown | 27 (11)  28 (11)  192 (78)  31 | 8 (7)  13 (11)  96 (82)  22 | 19 (15)  15 (12)  96 (74)  9 | 0.14 |
| ECOG status (n, %)  0/1  2/3  Unknown | 235 (86)  38 (14)  5 | 125 (93)  9 (7)  5 | 110 (79)  29 (21)  0 | <0.001 |
| mCCI Score (n, %)  0  1+  Unknown | 176 (65)  93 (35)  9 | 88 (66)  45 (34)  6 | 88 (65)  48 (35)  3 | 0.90 |
| Paraneoplastic Syndrome (n, %)  No  Yes  Unknown | 253 (93)  19 (7)  6 | 124 (93)  9 (7)  6 | 129 (93)  10 (7)  0 | 1.00 |
| Pre treatment brain imaging (n, %)  CT  MRI  None  Unknown | 75 (28)  174 (65)  17 (6)  12 | 56 (44)  58 (45)  14 (11)  11 | 19 (14)  116 (84)  3 (2)  1 | <0.001 |
| Radiotherapy Dose  40Gy/15  45Gy/30 BID  60Gy/30  66Gy/33  Other | 183 (66)  82 (29)  3 (1)  3 (1)  7 (3) | 134 (96)  0 (0)  0 (0)  0 (0)  5 (4) | 49 (35)  82 (59)  3 (2)  3 (2)  2 (1) | <0.001 |
| Chemotherapy (n, %)  Concurrent  Sequential  Unknown | 230 (83)  46 (17)  2 | 111 (81)  26 (19)  2 | 119 (86)  20 (14)  0 | 0.34 |
| Brain Surveillance (n, %)  MRI  No-MRI | 155 (56)  123 (44) | 56 (40)  83 (60) | 99 (71)  40 (29) | <0.001 |
| Brain Relapse Salvage Therapy (n, %)*  WBRT  SRS | 58 (88)  8 (12) | 32 (97)  1 (3) | 26 (79)  7 (21) | 0.054 |

**Table S1: Patient characteristics associated with diagnosis date; before median date of diagnosis (historical) and after median date of diagnosis (contemporary).**

Abbreviations: ECOG, Eastern Cooperative Oncology Group; PCI, prophylactic cranial irradiation; mCCI, modified Charlson Comorbidity Index; WBRT, whole brain radiotherapy; SRS, stereotactic radiosurgery

*Only patients with brain relapse and received salvage therapy were included
